# Biological, Chemical, and Nutritional Food Risks and Food Safety Issues From Italian Online Information Sources: Web Monitoring, Content Analysis, and Data Visualization

**DOI:** 10.2196/23438

**Published:** 2020-12-14

**Authors:** Barbara Tiozzo, Mirko Ruzza, Valentina Rizzoli, Laura D'Este, Mosè Giaretta, Licia Ravarotto

**Affiliations:** 1 Department for Training, Communication and Support Services Istituto Zooprofilattico Sperimentale delle Venezie Legnaro Italy; 2 IT Service Istituto Zooprofilattico Sperimentale delle Venezie Legnaro Italy

**Keywords:** big data, online information sources, web monitoring, content analysis, data visualization, food risks, risk communication

## Abstract

**Background:**

With rapid evolution of the internet and web 2.0 apps, online sources have become one of the main channels for most people to seek food risk information. Thus, it would be compelling to analyze the coverage of online information sources related to biological, chemical, and nutritional food risks, and related safety issues, to understand the type of content that online readers are exposed to, possibly influencing their perceptions.

**Objective:**

The aim of this study was to identify the types of online sources that are predominantly covering this theme, and the topics that have received the most attention in terms of coverage and engagement on social media.

**Methods:**

We performed an analysis of big data related to food risks by combining web monitoring techniques, content analysis, and data visualization of a large amount of unstructured text. Using a dictionary-based approach, a web monitoring app was instructed to automatically collect web content referring to the food risk and safety field. Data were retrieved from March 2017 to February 2018. The validated corpus (N=12,163) was subject to automatic and manual content analysis. Results were combined with descriptive statistics extracted from Web-Live and processed with Qlik Sense.

**Results:**

Nutritional risks and news about outbreaks, controls, and alerts were the most widely covered topics. Thematic sources devoted major attention to nutritional topics, whereas national sources covered food risks, especially during food emergencies. Regarding engagement on social media, readers’ interest was higher for nutritional topics and animal welfare. Although traditional sources still publish a great amount of content related to food risks and safety, new mediators have emerged as alternative sources for food risk information.

**Conclusions:**

This mixed methodological approach was demonstrated to be a useful means for obtaining an accurate characterization of the online discourse on food risks, and can provide insight into how the monitored sources contribute to the process of risk communication.

## Introduction

### Background

Risk perception and communication research has shown that many consumers worry about the quality and safety of the food they buy and eat [1-3]. To combat this concern, consumers actively engage in food risk information-seeking to gain useful recommendations about how to prevent and potentially face these risks [4].

Although television remains the most common source of information about food risks [3,5], online sources (ie, the internet and digital media) have become some of the main channels of searching for food risk information for most people [6-9]. The internet offers a wide variety of sources for health searches. In addition to news websites, many other forms of online health communities serve as online information sources [7,10,11], including search engines, health websites, social networking sites such as Facebook and Twitter, and social question and answer services, where risk information is permanently available and accessible [12].

Since information on food risks is widely sought online, it would be compelling to analyze the coverage of food risk by online information sources. According to previous literature, assessing the quantity of information about food risks that is available online can be of paramount importance to estimating how much information people may be exposed to and the extent to which people might engage with that information, possibly becoming an amplification station in terms of risk perception [13-15].

Web monitoring methodologies and techniques represent some of the best ways to analyze how much and where a topic is represented on the internet. Web monitoring is a set of specific activities that are planned to monitor and collect text from online sources to gain a better understanding of, and to measure the extent, time, and content that people are talking about with respect to a topic, person, brand, product, or service [16-19]. Monitoring what is being said online provides opportunities and benefits in terms of competitive analysis; sentiment and opinion analysis; knowledge discovery; consumer knowledge management; management and decision-making processes; social media strategies; creation or innovation of products and services; and predictions of scenarios, trends, and events [20].

As an application of text mining techniques [21,22], the web monitoring process basically consists of techniques for collecting, extracting, analyzing, and presenting online data that are relevant to the practitioner’s research aims [23]. For example, using a dictionary-based approach [24,25], the internet environment is scanned to gather relevant text according to a predefined taxonomy that determines the content that has to be automatically collected using, for example, application programming interfaces and/or crawlers. The selected content is then extracted and analyzed to discover patterns or relationships, which can be translated into valuable information [26-28].

Although web monitoring and text mining are a strong tradition in corporate communication [16,18,19,29,30], their application is a novelty in the domain of food risk communication. To the best of our knowledge, this study represents a first attempt to evaluate the online coverage of food risks using a big data approach.

### Study Aims

This study was designed to analyze big data related to food risks by combining web monitoring, content analysis, and data visualization approaches to determine the type of information Italian online sources provide about food risks and safety. Specifically, we addressed the following research questions:

RQ1: Which online information sources have covered food risks the most and received major engagement on social media?

RQ2: Which food risk topics have received major attention in terms of coverage and engagement on social media?

RQ3: Is there any difference in the coverage of food risk and safety topics among the monitored sources?

Based on these questions, four main topic categories were considered: biological, chemical, nutritional risks, and food safety issues. Biological risks include all hazards caused by bacteria, parasites, and viruses that can lead to food spoilage and possibly food poisoning for the consumer [31], such as *Salmonella* and *Campylobacter*. Chemical risks are hazards to human health that are derived from atoms, molecules, or substances, and can be present in raw ingredients for many reasons or can be formed in food during the production chain or the managing of food by the consumer after purchase [31-33]. A nutritional risk refers to the likelihood of adverse events occurring as a result of both poor nutritional quality and the amount of food consumed. This risk is associated with exposure to an inadequate diet, over or under in terms of quality and amount, for a certain period. Discourses on these risks belong to the broader concept of food safety that goes beyond the single risk agent and generally refers to all practices that are used to keep food safe, including cultural, scientific, and economical aspects, through the involvement of several different figures mandated to maintain food safety throughout the food chain.

## Methods

### Phase 1: Development of the Monitoring Profile

**Figure 1:**
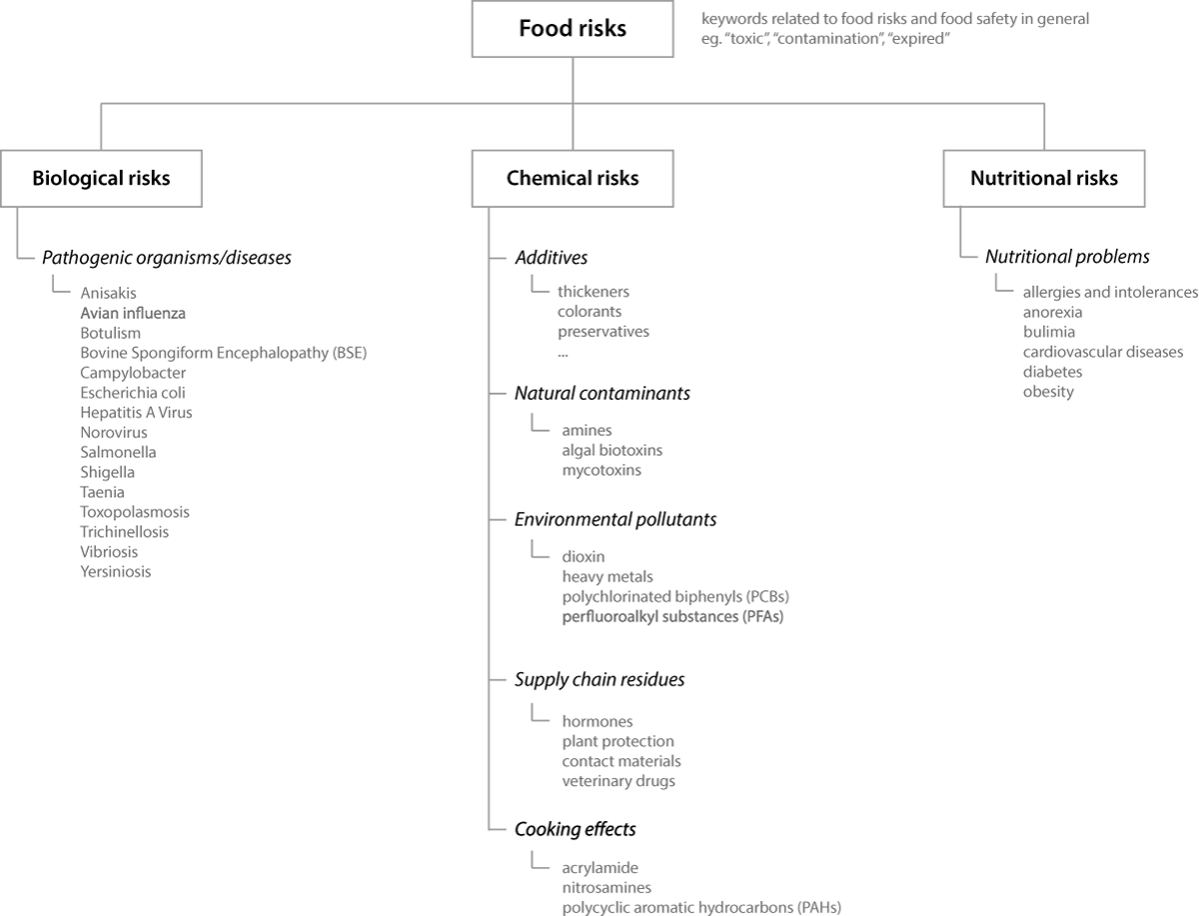
Logical scheme of the monitoring profile.

According to the logical scheme shown in Figure 1, a list of terms and rules referring to the four main categories under study was defined. Two of the authors, being senior experts with long-term experience in food risk management, identified the terms that were further validated by an external senior expert. The validated terms served as a starting point to define the monitoring profile, which is shown in Multimedia Appendix 1.

### Phase 2: Data Acquisition (Web Monitoring)

Data were collected from March 1, 2017 to February 28, 2018 using Web-Live, a web monitoring app [22] (for further details, see Multimedia Appendix 2) that was instructed to work according to the monitoring profile. Every day throughout the reference period, the content automatically collected the day before was listed in descending order with respect to the number of estimated views. The list was read and analyzed to select the content characterized by greater visibility and higher relevance in terms of food risk significance. This selection and validation process was performed in Web-Live according to the following procedure: based on the reading of the title, snippet, or complete text of the content, content that was not relevant to the topic of food risks was excluded; only content assessed as relevant was included in the final corpus, up to a pre-established limit of 50 items per day; and each day, a preliminary tag was assigned to each piece of daily validated content to track the main topic it discussed and to inform further content analysis procedures (first level of tagging).

### Phase 3: Data Analysis and Interpretation

After the monitoring phase, the content and statistics related to the validated corpus were extracted from Web-Live.

To answer RQ2, we first performed an automated content analysis. Automatic content analysis procedures based on text mining techniques have gained importance and popularity in the digital media environment due to the presence of larger datasets [34-36], and these methods have already successfully been used to analyze text that refers to food risks and safety issues [37-40]. A subcorpus composed of all validated content without content published on social media was extracted. Texts from social media were excluded because these types of texts differ in terms of length (they are normally shorter) and linguistic register. The resulting subcorpus was subject to a preprocessing phase [41] using TaLTaC2 software version 2.10.2 [42]. By means of the Reinert method, implemented in the R interface Iramuteq (version 0.7) [43], clusters of words referring to the same class of meaning (ie, lexical worlds) [44] were individuated to reveal patterns that characterize the online content on food risks. The associations among the clusters and the presence of the clusters over time were then observed using R software v. 3.5.1.

The results from automated content analysis guided the manual qualitative analysis of the retrieved content [45]. According to an open coding process [46], a label was assigned to each content in the corpus (second level of tagging). Starting from the lexical words identified by the automatic analysis and according to the tags that were preliminarily associated with the 50 pieces of daily validated content (first level of tagging), we proceeded with drawing up a list of the tags in use (Multimedia Appendix 3). When a new topic emerged, the list of tags was reviewed and refined iteratively. If two or more content items referred to the same topic, the content was assigned the same tag. In this manner, new tags were added to the list as they were created, thereby facilitating the researcher’s task in assigning them without overlaps or duplication. Mutually exclusive tags were applied according to the prevalent topic treated in the content. These tags were then grouped into broader tags (third and fourth levels of tagging). The labeling process was performed separately by two trained coders using Microsoft Excel. Any discrepancies were discussed and resolved until an agreement was reached, and a third coder was involved in a supervisory role and guaranteed consistency in the tag assignment.

To answer RQ1, the sources with more than 10 content items were selected and subsequently grouped into a set of 6 source categories based on criteria such as editorial line, distribution on the territory, and type of author (Multimedia Appendix 4).

According to recent literature, data visualization is not only considered an output of the research process or a way of communicating results but is further considered to be an integral part of the research process itself, because one of its goals is to derive insight from massive, dynamic, ambiguous, and often conflicting data [47]. With this approach, statistics related to the validated corpus extracted from Web-Live were combined with topic and source categories resulting from the content analysis using the data visualization software Qlik Sense, which provided an overview of the distribution of content among the different sources and topics.

## Results

### Data Retrieval

The web monitoring phase returned 12,163 validated content items from the 209,847 raw data retrieved by the web monitoring app. These 12,163 validated items were considered the final corpus that was further analyzed. Overall, this content reached a total of 1,117,491 interactions. All of the text and the statistics related to the final analyzed sample are available in Multimedia Appendix 5 and in a dedicated web app [48].

### Online Information Sources Related to Food Risk and Safety Topics (RQ1)

As shown in Table 1, the greatest amount of content from the total corpus (N=12,163) was published on web sources and the remaining content was published on social media. Overall, the content was published by 3230 unique sources that were equally active both as web sources and social media accounts. Among these, 240 unique sources published more than 10 content items (classified sources), whereas 2990 sources did not reach the threshold of 10 (nonclassified sources). Therefore, almost half of the total content was published by a larger number of different sources on an occasional basis, while the other half was published by a limited number of sources, mainly websites. The content published on social media was notably more extensive for the nonclassified sources.

**Table 1:** Major sources based on amount of content and website/social media ratio.

Source type	Unique sources (N=3230)			Contents (N=12,163)		
	Total, n (%)	Websites, n (%)	Social media, n (%)	Total, n (%)	Websites, n (%)	Social media, n (%)
Classified sources	240 (7.43)	207 (86.3)	33 (13.7)	6474 (53.23)	5823 (89.94)	651 (10.06)
Nonclassified sources	2990 (92.57)	1431 (47.86)	1559 (52.14)	5689 (46.8)	3200 (56.25)	2489 (43.75)
All sources	3230 (100.00)	1638 (50.71)	1592 (49.29)	12,163 (100.00)	9023 (74.18)	3140 (25.82)

A specialized website about food safety issues (ilfattoalimentare.it) emerged as the top source. Other major sources of interest were about the weather, the environment, and health news media (eg, meteoweb.eu, greenme.it, greenstyle.it) (Table 2).

Among the classified sources (Table 3), the thematic sources devoted major attention to the topic, followed by generalist news sources, local sources, and national sources.

**Table 2:** Major sources by coverage.

Source name	Category	Contents, N
Ilfattoalimentare.it	Thematic source	256
Ansa.it	National source	250
Meteoweb.eu	Thematic source	194
It.blastingnews.com	Generalist news source	170
Affaritaliani.it	Generalist news source	125
Greenme.it	Thematic source	108
Repubblica.it	National source	107
Greenstyle.it	Thematic source	106
FB Il Fatto Alimentare	Thematic source	90
Lastampa.it	National source	87
Freshplaza.it	Thematic source	81
TW Studio ABR	Thematic source	73
Adnkronos.com	National source	67
Tio.ch	Local source	67
Mainfatti.it	Generalist news source	66

**Table 3:** Classified source types based on amount of content.

Source type (classified)	Contents, n (%) of the total corpus (N=12,163)
Thematic sources	2480 (20.39)
Generalist news sources	1304 (10.72)
Local sources	1240 (10.19)
National sources	916 (7.53)
Organizational sources	405 (3.33)
Alternative information sources	103 (0.85)
Other sources	26 (0.21)
Total	6474 (53.23)

**Figure 2:**
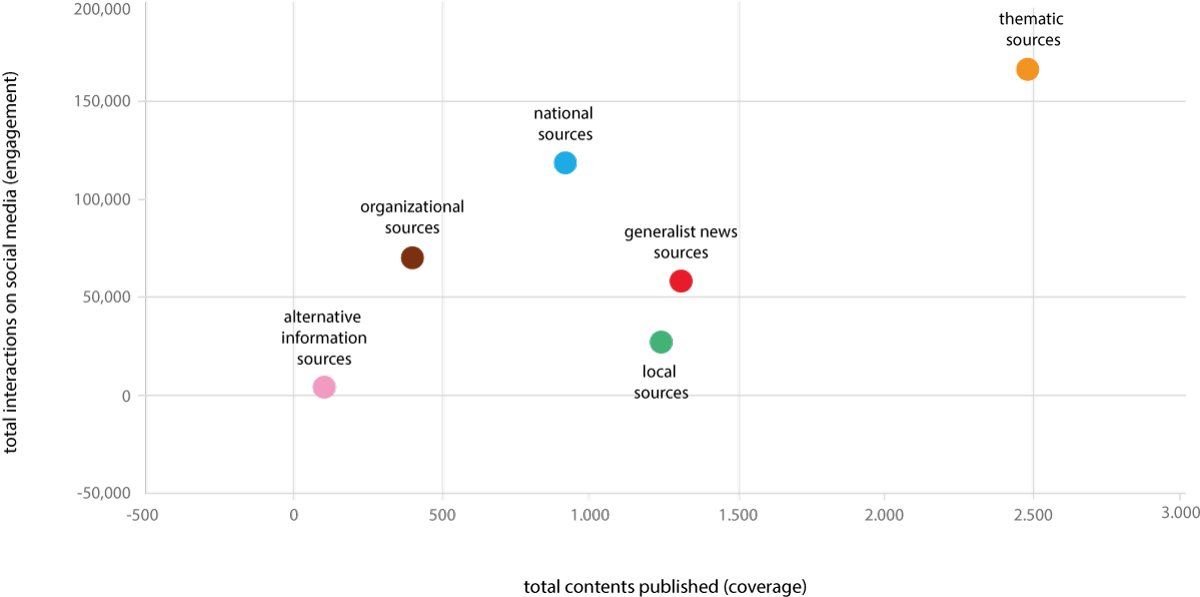
Content and engagement on social media for the classified sources.

Regarding the engagement on social media of the content published by the classified sources (Figure 2), the thematic sources achieved the highest number of interactions from online readers, whereas the number of interactions was limited for generalist news and local sources, despite the amount of published content from these sources. National sources ranked in second place, although they published less content related to food risks and safety.

### Food Risks and Safety Topics That Received Major Coverage and Engagement on Social Media (RQ2)

**Figure 3:**
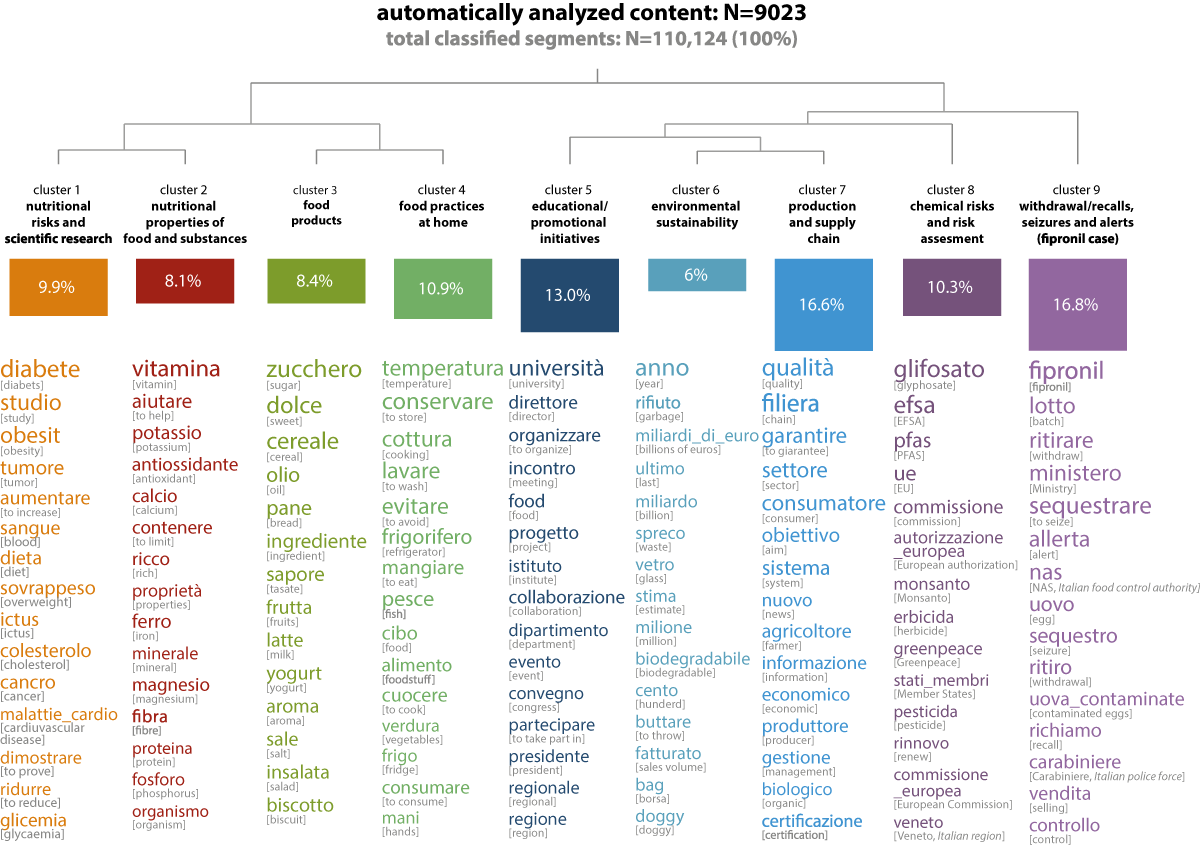
Topics and lexical words.

From the considered subcorpus (n=9023, 74.18% of the total corpus), the automated content analysis turned out nine clusters or lexical worlds (110,124 classified segments, representing 87.08% from the total of 126,463 segments; Figure 3). The hierarchical descending analysis divided these clusters into two subgroups of similar clusters based on content: the former (clusters 1-4) referred to nutritional and biological risks related to the consumption, manipulation, and conservation of food at home, whereas the latter (clusters 5-9) concerned chemical risks and more general or overarching food safety issues, with a strong focus on policies, regulations, and the activities of figures active in this field, such as authorities, companies, institutions, and associations. Multimedia Appendix 6 provides examples of the text segments for each cluster.

The manual procedure returned three levels of thematization of food risks in online sources: 45 in-depth categories (second level of tagging), 10 macrocategories (third level of tagging), and (2 macroareas (fourth level of tagging)

**Figure 4:**
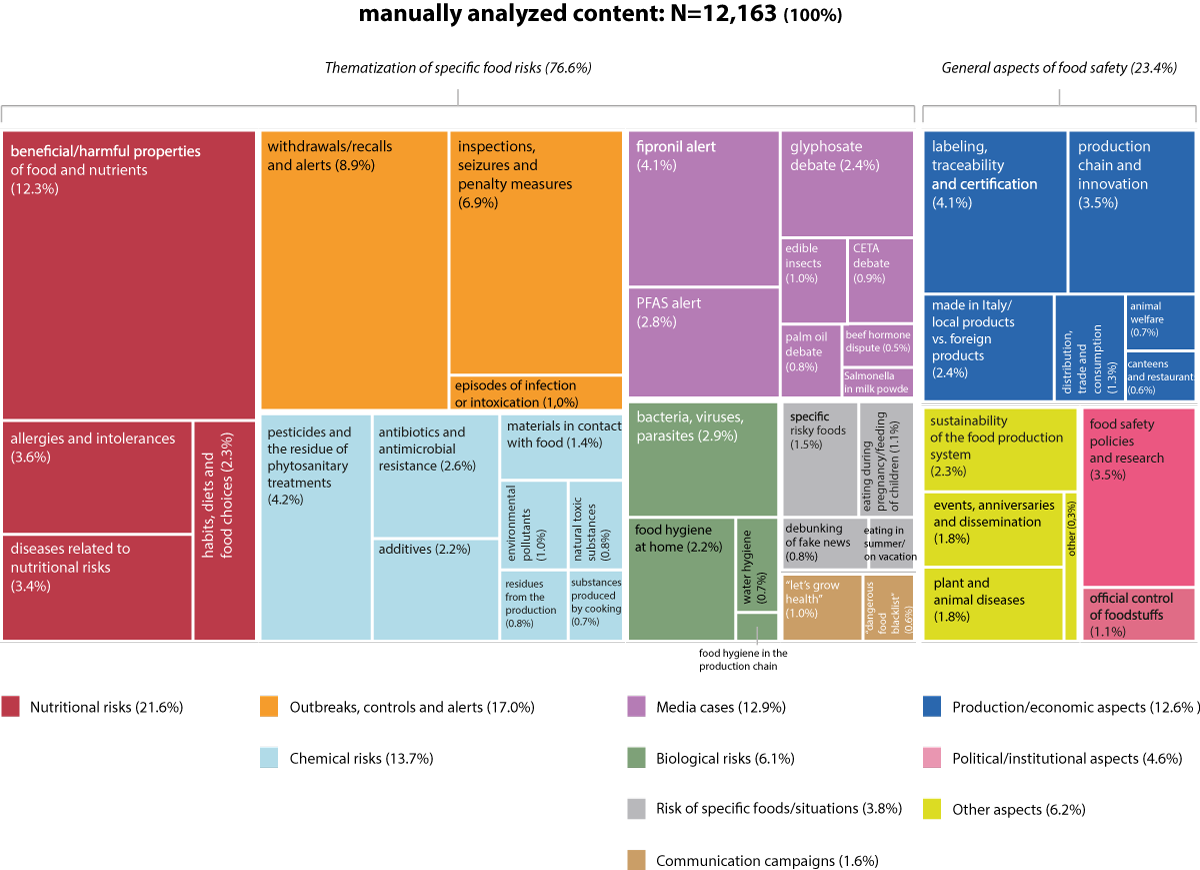
Content for each category identified by the manual content analysis.

The three levels of tagging were matched with the descriptive statistics provided by Web-Live using Qlik Sense to associate the amount of coverage and engagement on social media values with the identified levels of tagging, and to establish which topics emerged from the web monitoring representing food risk and safety issues in the Italian digital ecosystem (Figure 4).

The online coverage of food risks turned out to be composed of two main areas (fourth level of tagging): “thematization of specific food risks” and “general aspects of food safety,” which accounted for 76.61% (9318/12,163) and 23.39% (2845/12,163) of the corpus, respectively.

The macroarea “thematization of specific food risks” includes content referring to one or more food risks that stood out well to identified topics. The macroarea “general aspects on food safety” included content that generally refers to food safety as a public health problem, without mentioning or focusing on specific risks. This macroarea was dominated by issues related to the origin and traceability of food products, and the role of certifications and labeling as a means for the consumer to control their own safety of food. In much of this content, products coming from abroad are considered to be unsafe, whereas products of national and local origin are considered safe. This concept is mainly supported by the communications of Italian food producers and trade associations in the agricultural and livestock sector, but is also reinforced by news media and other figures such as politicians and consumer associations. Much of the content is also related to technological developments and innovations in the food production chain, as well as to regulatory policies passed by health authorities and public initiatives created by institutions and associations to ensure food safety.

The macrocategory “nutritional risks” (third level of tagging), with the highest amount of content, emerged as the most important topic associated with food risks, followed by “outbreaks, controls, and alerts.” A good level of coverage was also reached by content referring to “chemical risks” and “media cases.” The macrocategory “nutritional risks” mainly reports news about “beneficial/harmful properties of food and nutrients” (1497/2623, 57.07% of the content of the macrocategory). This content mentions project results from universities and research centers, focusing on the properties of foods, nutrients, diets, or specific eating habits. This content originates within the scientific community, and online sources translate it into practical advice for the consumer, sometimes reviewing the positive/negative properties of specific foods. Often, this content questions previous studies, highlighting the contradictions in experts’ opinions and the partiality of scientific knowledge. The macrocategory “outbreaks, controls, and alerts” reports content related mainly to “withdrawals/recalls and alerts” (1079/2064, 52.28% of the content of the macrocategory) and “inspections, seizures, and penalty measures” (862/2064, 41.76% of the content of the macrocategory). This content derives from the coverage of food alert notifications from the Rapid Alert System for Food and Feed (RASFF) of the European Union, as well as from news that follows the activities and measures adopted by the local health authorities that carry out official controls on the food production and distribution chain. The macrocategory “chemical risks” mainly reports content referring to “pesticides and the residue of phytosanitary treatments” (514/1671, 30.76% of the content of the macrocategory) and “antibiotics and antimicrobial resistance” (319/1671, 19.09% of the content of the macrocategory). Food risks are often associated with intensive farming methods and industrial production, showing a certain distrust of industry and technology. Conversely, the concept of safe and healthy food is associated with the concepts of naturalness and organic farming. The macrocategory “media cases” deals mainly with food alerts referring to the “fipronil alert” (493/1566, 31.48% of the content of the macrocategory), “PFAS (per- and polyfluoroalkyl substances) alert” (345/1566, 22.03% of the content of the macrocategory), and “glyphosate debate” (297/1566, 18.97% of the content of the macrocategory). These cases, and their developments and implications, are generally depicted by adopting the frame of a scandal. This content also importantly covers the public debate on risk management and regulatory policies; activities by risk assessors and managers such as European institutions, national or local health authorities, and private companies are discussed and juxtaposed, each of them feeding the debate with their own views on and perspectives of food risks and safety measures.

**Figure 5:**
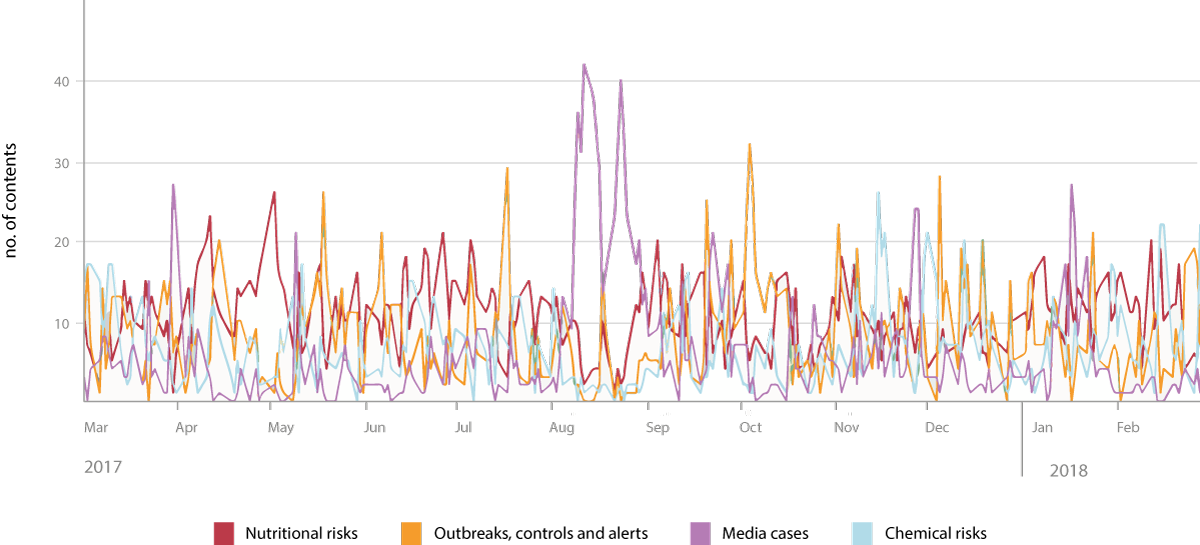
Trend of the content related to the major food risk topics.

Regarding the time distribution of the most commonly covered macrocategories (Figure 5), no significant differences in coverage were found. That is, the attention given by the monitored sources to such topics was rather stable across time, and these topics equally contributed to shaping the source agenda in the reference period. The only sharp increase in coverage was registered during August 2017, which corresponds to the fipronil alert that catalyzed the increased attention of the monitored sources [49], corresponding to a decrease in the coverage of the other major topics.

**Figure 6:**
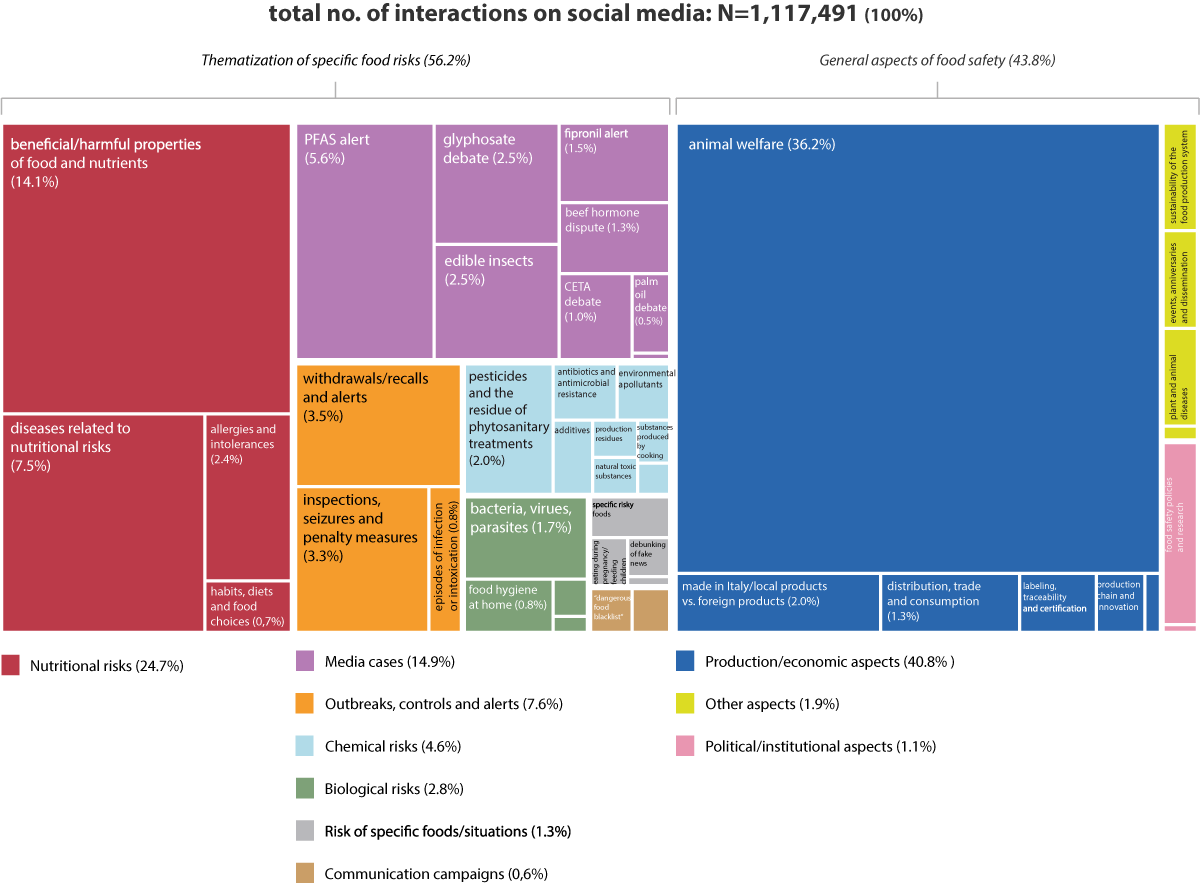
Social media interactions for each category identified by the manual content analysis.

**Figure 7:**
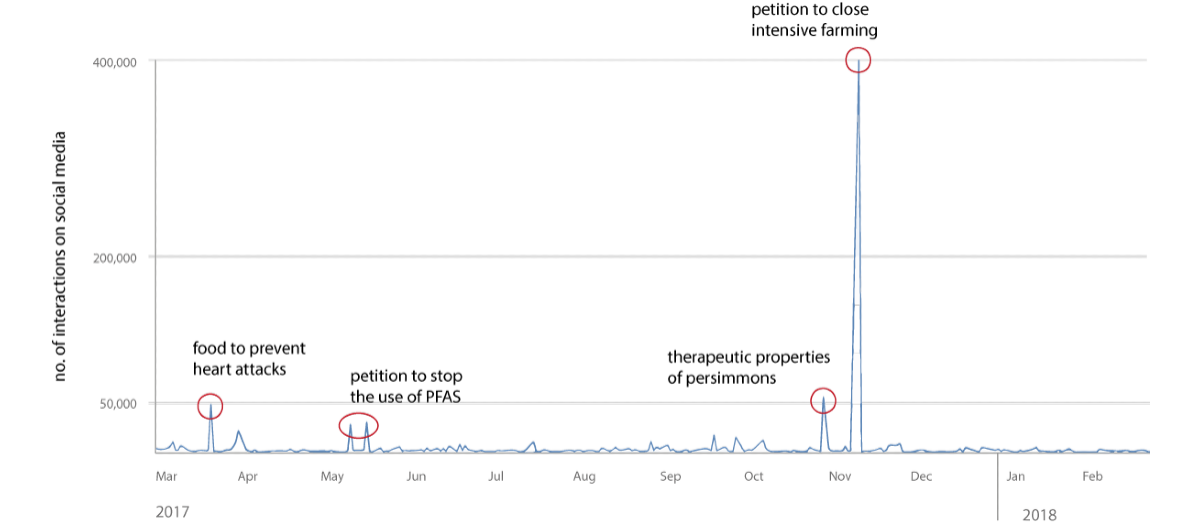
Trend of engagement on social media and major peaks.

Regarding engagement on social media (Figure 6), the greatest number of interactions (n=398,532) was recorded in November 2017 by a unique content item of the in-depth category “animal welfare” related to a petition promoted through the online portal change.org to support the closure of intensive farming. The other content items that gained a high number of interactions, albeit with significantly lower numbers, were as follows (Figure 7): (1) news content on foods considered to prevent heart attacks (48,704 interactions), published in March 2017, and belonging to the in-depth category “diseases related to nutritional risks”; (2) two distinct news articles published in May 2017 by the environmental association Greenpeace to promote their petition to stop the use of PFAS in the Veneto region (28,182 and 30,561 interactions, respectively) and belonging to the in-depth category “PFAS alert”; and (3) a news article talking about the therapeutic properties of persimmons (56,130 interactions) that was published in October 2017, and belonged to the in-depth category “beneficial/harmful properties of food and nutrients.”

### Distribution of the Coverage of Food Risk and Safety Topics Among the Monitored Sources (RQ3)

Matching the major topics of the third level of tagging with the amount of content published by the classified sources, we obtained the distribution of these topics by source and how they contributed to composing the source agenda (Table 4).

Thematic sources generally published content about “nutritional risks,” whereas generalist news sources and local sources mainly published news related to “outbreaks, controls, and alerts.” National sources and organizational sources provided more space to the coverage of “media cases.” Alternative information sources talked mostly about “chemical risks.”

**Table 4:** Most widely covered macrocategories and topics (third level of tagging) for each source category.

Source type		Contents, n (%)	
**Thematic source (N=2408)**			
	Nutritional risks		825 (33.26)
	Chemical risks		364 (15.12)
	Outbreaks, controls and alerts		301 (12.50)
	Production/economic aspects		259 (10.76)
	Media cases		196 (8.14)
	Biological risks		177 (7.35)
	Specific foods/situations		129 (5.36)
	Political/institutional aspects		104 (4.32)
	Other aspects		102 (4.32)
	Communication campaigns		23 (0.96)
**Generalist news sources (N=1304)**			
	Outbreaks, controls and alerts		331 (25.38)
	Nutritional risks		281 (21.55)
	Media cases		188 (14.42)
	Chemical risks		144 (11.05)
	Production/economic aspects		121 (9.28)
	Biological risks		77 (5.90)
	Other aspects		61 (4.68)
	Specific foods/situations		51 (3.91)
	Political/institutional aspects		29 (2.22)
	Communication campaigns		21 (1.61)
**Local sources (N=1240)**			
	Outbreaks, controls and alerts		315 (25.40)
	Media cases		274 (22.10)
	Production/economic aspects		160 (12.90)
	Nutritional risks		130 (10.48)
	Chemical risks		108 (8.71)
	Other aspects		92 (7.42)
	Political/institutional aspects		58 (4.68)
	Biological risks		49 (3.95)
	Specific foods/situations		43 (3.47)
	Communication campaigns		11 (0.88)
**National sources (N=916)**			
	Media cases		184 (20.1)
	Nutritional aspects		167 (18.2)
	Chemical risks		150 (16.4)
	Production/economic aspects		135 (14.7)
	Outbreaks, controls and alerts		85 (9.3)
	Other aspects		82 (9.0)
	Biological risks		37 (4.0)
	Political/institutional aspects		35 (3.8)
	Specific foods/situations		32 (3.5)
	Communication campaigns		9 (1.0)
**Organizational sources (N=405)**			
	Media cases		91 (22.5)
	Chemical risks		79 (19.5)
	Nutritional aspects		73 (18.0)
	Production/economic aspects		60 (14.8)
	Political/institutional aspects		31 (7.7)
	Other aspects		23 (5.7)
	Outbreaks, controls and alerts		20 (4.9)
	Biological risks		15 (3.7)
	Specific foods/situations		10 (2.5)
	Communication campaigns		3 (0.7)
**Alternative information sources (N=103)**			
	Chemical risks		36 (35.0)
	Nutritional risks		33 (32.0)
	Media cases		13 (12.6)
	Outbreaks, controls and alerts		9 (8.7)
	Biological risks		5 (4.9)
	Other aspects		3 (2.9)
	Production/economic aspects		3 (2.9)
	Specific foods/situations		1 (1.0)

## Discussion

### Principal Findings

The results of this study show that food risk and safety issues are widely covered by the monitored online sources. During the period under study, the sources continued publishing content and their interest in the theme was stable over time, apart from media peaks corresponding to major food scares. This finding resonates with previous results referring to the coverage of food risks by daily newspapers in Italy [13,40]. The results also show that the online sources do not talk about food risks and safety only during a crisis but, importantly, also during periods of peace, when a great part of the coverage was also tracked. This pattern enables online readers to realize the complexity of this field in terms of both the topics and the actors concerned, even when there is no imminent or direct risk for people.

### RQ1: Which Online Information Sources Have Covered Food Risks the Most and Received Major Engagement on Social Media?

Notwithstanding the growing role of social media as a source of information [6], a great amount of the content on food risk and safety issues is still published on websites and news portals, which differ in terms of source type and editorial line. Owing to the monitoring and analysis of content from a large set of sources, our data reveal that traditional sources (eg, national and local news media) have been joined by other types of sources that give voice to a variety of authors, such as thematic and generalist news sources. Indeed, food risk and safety issues are also covered by numerous sources that have published one or more items related to the topic in an occasional way and without making it a distinctive sign of their editorial line. This highlights how actors not primarily involved in the management and communication of food risks can actually play the role of alternative online science communicators. This result resonates with the increasingly consolidated phenomenon that sees activists and advocates in the health and environmental fields competing with other societal actors for public attention to issues [50], and scientists are not always seen by the relevant audience as figures who can be easily related to, especially in social media communication [51]. Our data about engagement on social media peaks also confirm this trend.

The thematic sources ranked first for the amount of published content on food risks and reached the highest level of engagement on social media. This is probably because these sources offer more specialized content on the subject, targeting the attention of readers with a specific interest in this content who are therefore more likely to share and discuss it with their social network. Importantly, local and national sources (mainly news media websites) still cover food risk and safety topics: authors belonging to national sources are among the most active in terms of published content, and their informative role is still greater in cases of food emergencies (“media cases” tag). Interestingly, the second type of source in terms of the amount of published content is online sources of generalist news. This category includes national news portals, news aggregator websites, citizen journalism platforms, and related social accounts, representing the change that news production practices have undergone in the last few decades [52]. A smaller amount of content was published by sources of alternative information that generally publish fake news or content of uncertain reliability. However, this content obtained almost no engagement on social media, thereby limiting the potential dissemination of inaccurate news.

### RQ2: Which Food Risk Topics Have Received Major Attention in Terms of Coverage and Engagement on Social Media?

The “food risk” theme consists of several smaller topics that can be divided into two major categories: “food risks in everyday life” and “food risk governance.” The former category discusses nutritional and biological risks that people can manage on their own and upon which they can have direct control by means of simple actions such as diet choices and food preparation practices. The latter category refers to national and international activities to practice food safety and to ensure that the debate about risk management and related policies is made accessible to the public. This latter category includes chemical risks that are generally perceived as not directly controllable in everyday life, as the responsibility for such risks is considered to be external and technology-driven.

The prevalence of content about nutrition resonates with the fundamental role that nutritional science has assumed in shaping food meanings and practices, and nutrition experts’ presence and advice are now prolific in the media [53]. The results show that this also occurs in the Italian online communication landscape, where nutritional information is widely covered by the monitored sources and has also gained considerable interest from readers, with the macrocategory “nutritional risks” being the topic with the second-highest value of engagement on social media. Indeed, this topic is very close to consumers’ everyday lives and possibly fulfills their information needs. Notwithstanding, the way this topic is discussed (ie, either corroborating or questioning previous research in this field) prevents readers from easily interpreting the information and, consequently, appropriately managing the risk itself in everyday life because they receive contradictory messages over time.

The significant number of mentions referring to the macrocategory “outbreaks, controls, and alerts” proves that news on withdrawals, recalls, inspections discovering a lack of safety measures, and foodborne outbreaks is regularly published online but normally does not receive particular amplification in terms of coverage and readers’ interactions. This content enters the national sources agenda when factors such as a high number of people concerned and the presence of victims or the unknown/new nature of the risk intervene to increase the newsworthiness of the event. This mechanism actually characterizes the macrocategory “media cases” and its related in-depth categories such as the “fipronil alert” [49]. This finding reinforces the role of external factors in influencing the newsworthiness and the coverage of risks, notwithstanding the characteristics of the hazards themselves.

The widespread interest of the monitored sources in “chemical risks” matches with what consumers report as their major concerns about food [3,54], which has also been explored in the Italian context [2]. Actually, the “media cases” that received major coverage had chemical origins and referred to pesticides (fipronil and glyphosate) and environmental pollutants (PFAS).

Despite the diffusion of foodborne diseases at home [55] and the need to help consumers prevent them [56-58], the results show that “biological risks” receives minor coverage compared to nutritional and chemical risks. Differences in the nature of these risks justify this coverage pattern. Biological risks generally refer to a consolidated knowledge of pathogens and related foodborne diseases, with well-defined instructions to prevent them. In contrast, chemical risks cannot be so easily perceived and managed, and their controversial nature makes them more attractive to cover. Indeed, the way these risks are reported gives rise to the many, often contradictory, opinions supported by the diverse figures involved (eg, risk assessors, risk managers, health authorities, consumer associations). Biological risks offer much fewer possibilities for using contradictory opinions to report facts, at least in the reference period of this study, in which no significant food crisis with biological origins occurred.

### RQ3: Is There Any Difference in the Coverage of Food Risk and Safety Topics Among the Monitored Sources?

The results further highlight some variability among the agendas of the monitored sources. National and organizational sources generally cover food risk topics when they are “media cases”: the news-making criteria for these sources is thus the occurrence of “risk events” [14], where the story can be featured in terms of scandal, emergency, and victims, and generally goes beyond local boundaries. Despite the lower amount of this content, it reached a good level of engagement on social media. This means that people are more likely to interact with content referring to major national events that possibly refer to a threat to public health. Generalist news sources and local sources give more space to issues related to “outbreaks, controls, and alerts”: these issues generally originate as local facts that occur in well-defined territories, thus justifying the widespread interest of local sources in this type of content.

Previous studies focusing on the coverage of food risks by online daily newspapers showed that the great majority of RASFF data did not receive specific mention, apart from food alerts [40]. Notably, this study not only confirms the preference of national sources for “food risk events” but also shows that a great number of alerts mentioned in the RASFF or more general content referring to official controls on food by health authorities are actually mentioned by online thematic, generalist, and local sources through which people can come to know about such information.

### Limitations

A first important limitation of this study refers to the inherent nature of big data that influences digital social research in terms of sample representativeness and access [35]. Our data were retrieved by a commercial web-monitoring app, whose algorithms are not completely disclosable for reasons of commercial protection [22]. The sampling method also suffered from access restriction from social media platforms, which give only partial access to their data [35,59]. To overcome these limitations and solve any opacity, we included the monitoring profile used to retrieve the analyzed content (Multimedia Appendix 1). Notably, the tracking of content related to the fipronil alert proved the sensitivity of the monitoring profile and the selection procedure, as the keyword “fipronil” was not included in the monitoring profile. In addition, the results need to be interpreted and contextualized within the limit of the 50 validated daily mentions: a different limit might have been liable to generate different coverage patterns.

The analysis of the web-monitoring data texts was performed with both automated and manual content analysis to obtain a deeper interpretation. We believe that both procedures are needed to better translate texts into sound and actionable information. The automated analysis enabled us to give robustness to the manual procedure: it provided us with the main branches referring to food risks and safety that worked as a starting point to guide the manual procedure. In turn, although it is a highly time-consuming activity, the manual analysis yielded detailed insights, and allowed us to understand the granularity and faceted nature of food risk and safety communication.

### Conclusions and Further Research

The combination of web monitoring, content analysis, and data visualization techniques proposed in this study was proven to be a viable approach to understand when the media pick up on an issue that does not actually jeopardize public health but generates a great deal of coverage, as well as where and from whom there is silence during an important issue or outbreak. The presence or lack of coverage of specific risks and topics in the online communication of food risks, as an output of web-monitoring data, can help communication practitioners in health agencies better hone their communication strategies and interventions.

The monitoring of a broad spectrum of online sources allowed for the tracking and interpretation of the interplay between science and society for a wider understanding of the mechanisms underlying food risk communication. This study demonstrates how the online information ecosystem of risk communication has changed and assisted with a proliferation of information sources that work as new mediators [60], the reliability and standing of which might sometimes be questionable. It is very clear that there is room for expert figures (eg, governmental institutions, food safety agencies, health authorities, research centers) to deliver food risk information using the online environment. Finally, communication practitioners could work in a closer relationship with the editorial offices of thematic sources, which talked about food risks more than the other types of sources. In this way, the scientific community could have greater visibility and coverage in the online environment, guaranteeing scientifically sound information.

## Appendix

Multimedia Appendix 1 Rules of keywords and logical operators used to configure the web monitoring app Web-Live [22] to monitor food risks and food safety topics in the Italian online media.

Multimedia Appendix 2 Description of the web monitoring app Web-Live.

Multimedia Appendix 3 Definition of the topic categories identified with the manual content analysis.

Multimedia Appendix 4 Categories of online information sources.

Multimedia Appendix 5 Texts and statistics related to the final validated and analyzed sample.

Multimedia Appendix 6 Examples of text segments for each cluster in the food risk corpus.

## Abbreviations

PFAS: per- and polyfluoroalkyl substances
RASFF: Rapid Alert System for Food and Feed
