# Supplementary material for: Biological, Chemical, and Nutritional Food Risks and Food Safety Issues From Italian Online Information Sources: Web Monitoring, Content Analysis, and Data Visualization
Source: J Med Internet Res. 2020 Dec 14;22(12):e23438. doi: 10.2196/23438 (PMC7769687; doi:10.2196/23438)
Supplement: Multimedia Appendix 2 [file jmir_v22i12e23438_app2.docx]

**Multimedia appendix 2. The Web-Live® application**

Online content was monitored and acquired using Web-Live^®^, a web monitoring application developed by the Italian company Extreme SRL (<http://www.web-live.it>). Web-Live^®^ automatically collects and classifies online content published in 43 different languages.

The application retrieves content and data from the web according to a monitoring profile, i.e., a system of rules based on a combination of keywords and logical operators. These rules are used to query search engines (Google, Bing, Yahoo) and social network websites (Facebook, Twitter, Google+, YouTube, Instagram) to retrieve relevant content and calculate different indicators for each content based on an integration with APIs, proprietary crawling algorithms and the combination with data by other data providers (e.g., Alexa, SEMRush, Similarweb).

In particular, Web-Live^®^ does the following:

- collects and indexes the text and different metadata of the web content that is relevant to the defined monitoring profile (e.g., the URL, a cached copy of the text, the publication date, the source in which it was published);
- calculates indicators related to the relevance of the source (number of monthly unique users estimated for websites, blogs and forums; number of followers for social accounts) where the content was published;
- calculates indicators referring to the diffusion (number of views) and the level of interaction (sum of the number of likes, comments and shares on social media) obtained from each content; and
- provides statistics and quantitative data relating to the collected content (e.g., amount of content produced by a source, time series of the content retrieved).

These data are saved in a database that can be accessed and searched through an online interface, which allows the user to filter content and relative quantitative statistics according to:

- different metadata (e.g., data, source, author, views, engagement);
- additional keywords (i.e., using the text research box to perform queries); and
- specific time span.

All processed data and statistics can be exported to spreadsheets.
