# Supplementary material for: Biological, Chemical, and Nutritional Food Risks and Food Safety Issues From Italian Online Information Sources: Web Monitoring, Content Analysis, and Data Visualization
Source: J Med Internet Res. 2020 Dec 14;22(12):e23438. doi: 10.2196/23438 (PMC7769687; doi:10.2196/23438)
Supplement: Multimedia Appendix 3 [file jmir_v22i12e23438_app3.docx]

**Multimedia appendix 3. Definition of the topic categories identified with the manual content analysis**

| CATEGORIES OF THE MAIN AREA “THEMATISATION OF SPECIFIC FOOD RISKS” (CATEGORY LEVEL 4) | | | | |
| --- | --- | --- | --- | --- |
| *Category level* *3* | *Category level 2* | | | *Description* |
| *Nutritional risks* | **Beneficial/harmful properties of food and nutrients** | | content talking about the healthy or beneficial properties of specific foods (e.g. beef, milk, salt, ginseng, spices) or substances and nutrients (e.g. sugars, fats, magnesium, potassium, selenium…) | |
|  | **Allergies and intolerances** | | content talking about substances that cause allergies and intolerances (e.g. nickel, hydrolysate, natural antihistamines, lactose, gluten…) | |
|  | **Diseases related to nutritional risks** | | content that mainly focus on health problems and connections with nutritional aspects are highlighted (e.g. cholesterol, hypertension, glycaemia, obesity, cardiovascular diseases, tumors, thyroid problems, anorexia, bulimia, fatty liver…) | |
|  | **Habits, diets and food choices** | | content that focus on the nutritional aspects of particular diets and food choices (e.g. Mediterranean diet, vegeterianism, veganism, junk food), feeding habits (e.g. breakfast, lunch, dinner) or nutrition related to different lifestyles (e.g. sedentariness, practising sports, etc) | |
| *Chemical risks* | **Additives** | | content talking about health risks, regulation and use of additives in food production (e.g. colourants, preservatives, antioxidants, nitrosamines, sulphites, anti-agglomeration agents, emulsifiers, enzymes, glutamate, endocrine disruptors) | |
|  | **Antibiotics and antimicrobial resistance** | | content talking about the health risks caused by antibiotic residues and the problem of antibiotic resistance | |
|  | **Pesticides and residues of phytosanitary treatments** | | content talking about health risks, regulation and use of pesticides and fertilizers (e.g. neonicotinoids, nitrates, organophosphates, copper, azadiractin, copper sulfate) | |
|  | **Residues from the production process** | | content talking about health risks, regulation and use of various substances and technologies in food production (e.g. veterinary drugs, hormones, feed, GMOs, furan, formaldehyde) | |
|  | **Environmental pollutants** | | content talking about health risks, regulations and accidents caused by environmental pollutants (e.g. heavy metals, hydrocarbons, PCBs, dioxins, radioactive substances) | |
|  | **Natural toxic substances** | | content talking about toxic substances that can be naturally produce by foods themselves (e.g. histamine, mycotoxins, aflatoxins, marine biotoxins, hydroxyantracene, pyrrolizidine alkaloids, thallium, arsenic) | |
|  | **Food contact materials** | | content talking about health risks derived from materials in contact with food (e.g. bisphenol, aluminum, phthalates, packaging materials, pot and pan materials) | |
|  | **Substances produced by cooking** | | content talking about acrylamide and IPA, both as products of cooking at the home and as of residues of the production system | |
| *Biological risks* | **Bacteria, viruses and parasites** | | content talking about risks related to the presence in food of specific bacteria, viruses and parasites (e.g. anisakis, botulin, Campylobacter, Hepatitis A, Hepatitis E, Escherichia coli, listeria, salmonella, BSE, bacillus cereus, staphylococcus, trichinella) | |
|  | **Food hygiene at home** | | content talking about risks caused by microorganisms (mentioned with generic terms in the retrieved content) during the purchase, storage, preparation and consumption of food at home | |
|  | **Food hygiene in the production chain** | | content talking about risks caused by microorganisms (mentioned with generic terms in the retrieved content) during the production and about food hygiene in stores and restaurants | |
|  | **Water hygiene** | | content talking about legionella or microorganisms (mentioned with generic terms in the retrieved content) living in aqueduct waters or in bathing areas | |
| *Media cases* | | **Fipronil alert** | content talking about the health alert at European level due to the presence of fipronil in eggs and egg products. | |
|  |  | **Pfas alert** | content talking about the health alert for environmental pollution from perfluoroalkyl substances that occurred in the province of Vicenza (a town in the North East of Italy) | |
|  |  | **Glyphosate debate** | content reporting information and the debate on the use of glyphosate and the realted risks for health, in particular related to the EFSA opinion and the EU legislation on the use this pesticide | |
|  |  | **Palm oil debate** | content reporting information and the debate on the use of palm oil and related risks for health | |
|  |  | **CETA debate** | content reporting information and the debate on the economic and food security implications of the free trade agreement between the European Union and Canada | |
|  |  | **Beef hormone dispute** | content reporting information and the debate on the US’ and President Trump's willingness to introduce customs duties, in response to the EU blockade of meat produced in the US because of the presence of hormones in such meat | |
|  |  | **Edible insects** | content reporting information and the debate on regulatory changes at European level regarding the sale of insects as novel foods | |
|  |  | **Salmonella in milk powder** | content talking about the health alert at European level due to the presence of Salmonella in some batches of milk powder | |
| *Risks of specific foods/situations* | | **Specific risky foods** | content talking about the risks from the consumption of particular foods (e.g. mussels, honey, milk, pasta, fish, raw milk, raw meat, raw fish, mushrooms ...), often overall dealing with both biological / chemical / nutritional aspects | |
|  |  | **Nutrition during pregnancy/feeding of children** | content talking about food risks during pregnancy, breast feeding or the growth of children | |
|  |  | **Eating in summer/on vacation** | content talking about food risks during summer and eating out on travel | |
|  |  | **Debanking of fake news** | content that denies fake or unfounded news about health alert or food risks related to particular foods | |
| *Outbreaks, controls and alerts* | | **Withdrawals/recalls and alerts** | RASFF notifications, withdrawals / recalls, warning from national Ministries of Health or European health authorities relating to the circulation of risky foods | |
|  |  | **Inspections, seizures and penalty measures** | interventions of local health authorities or law enforcement agencies by means of penalty measures, seizures and / or the closing of points of sale due to hygienic reasons or food frauds | |
|  |  | **Episodes of infection or intoxications** | news about hospitalizations or about people who have been sick as a result of eating contaminated or spoiled food | |
| *Communication campaigns* | | **“Let’s grow health” campaign** | A communication campaign by "Coop", one of the major operators of the Italian large-scale retail trade, to promote a line of poultry products that have been bred with a particular attention to the respect of animal welfare, and associated with greater level of safety as no antibiotics are used | |
|  |  | **"Dangerous foods black list" campaign** | A communication campaign by "Coldiretti", the main trade association of Italian agricultural producers, to discover the blacklists of foodstuffs dangerous to health that have been produced abroad but are present in the Italian market | |

| CATEGORIES OF THE MAIN AREA “GENERAL ASPECTS ON FOOD SAFETY” (CATEGORY LEVEL 4) | | | | |  |
| --- | --- | --- | --- | --- | --- |
| *Category level* *3* | *Category level 2* | | | *Description* | |
| *Political/*  *institutional aspects* | | **Food safety policies and research** | content mentioning the promotion of food safety by means of legislative interventions, scientific research activities, projects and institutional initiatives | |  |
|  |  | **Official control of foodstuffs** | content describing the control system on food and along the supply chain (i.e. official controls, self-control system), and results achieved on control activities (e.g. reports by the Ministry of Health, Ministry of Agriculture, EFSA, EU) | |  |
| *Production/*  *economic aspects* | | **Labeling, traceability and certifications** | content focusing on problems and opportunities for food safety linked to the certification of the origin and the quality of food | |  |
|  |  | **Made in Italy/local products vs foreign products** | content describing “made in Italy” and “km zero" food products as safe ones and / or products from abroad as risky foodstuffs | |  |
|  |  | **Production chain and innovation** | content dealing with zootechnical, agrotechnical and technological innovation issues in production systems related to food safety (e.g. selection of races, genetic interventions, food control systems along the supply chain, etc.) | |  |
|  |  | **Distribution, trade and consumption** | content mentioning food safety in news related to large-scale retail trade, retail markets, local markets, promotion and online commerce of food products, or presenting data on food consumption and consumers’ preferences | |  |
|  |  | **Canteens and restaurants** | content mentioning food safety in news relating to canteens and the food service sector | |  |
|  |  | **Animal welfare** | content mentioning food safety in relation to the issue of animal welfare on farms and in the production chain | |  |
| *Other aspects* | | **Sustainability of the food production system** | content mentioning food safety with a particular focus the ecological and social sustainability of the food production system (e.g. food security, waste reduction, packaging pollution, environmental impact and climate change | |  |
|  |  | **Events, anniversaries and dissemination** | content mentioning food safety in relation to recurring events and celebrations dedicated to food or food safety (e.g. world days, themed days), or in relation to initiatives for dissemination and consumer education (e.g. corporate campaigns, launches of book or tv programmes, etc) | |  |
|  |  | **Plant and animal diseases** | content mentioning food safety as part of news or in-depth information on animal or plant diseases associated with zoonotic risks (e.g. avian flu, CWD, African swine fever, blue tongue, animal poisoning, xylella) | |  |
|  |  | **Other** | other content mentioning food safety in general or more food risks, but without detailing associated risks for health and / or everything that does not fall into the previous categories | |  |
