# Supplementary material for: Biological, Chemical, and Nutritional Food Risks and Food Safety Issues From Italian Online Information Sources: Web Monitoring, Content Analysis, and Data Visualization
Source: J Med Internet Res. 2020 Dec 14;22(12):e23438. doi: 10.2196/23438 (PMC7769687; doi:10.2196/23438)
Supplement: Multimedia Appendix 4 [file jmir_v22i12e23438_app4.docx]

**Multimedia appendix 4. Categories of online information sources**

| Category of source | Description of the source |
| --- | --- |
| *National sources* | Websites and social media accounts of national press agencies and news media outlets (e.g. newspapers, tv and radio programmes) |
| *Local sources* | Websites and social media accounts of news and newapapers media outlets devoted to the diffusion of territory-based information (city/regional focus) |
| *Generalist news sources* | National news portals, news aggregator websites, citizen journalism platforms and related social media accounts |
| *Thematic sources* | Websites and social media accounts devoted to the in-depth coverage of specific topics (e.g. food, health, environment) |
| *Organizational sources* | websites and social accounts of companies, institutions, associations, political parties, research institutions |
| *Sources of alternative information* | news websites, information portals and social accounts that publish fake news or content of uncertain reliability |
| *Other sources* | Other types of sources |
