# Supplementary material for: Biological, Chemical, and Nutritional Food Risks and Food Safety Issues From Italian Online Information Sources: Web Monitoring, Content Analysis, and Data Visualization
Source: J Med Internet Res. 2020 Dec 14;22(12):e23438. doi: 10.2196/23438 (PMC7769687; doi:10.2196/23438)
Supplement: Multimedia Appendix 6 [file jmir_v22i12e23438_app6.docx]

**Multimedia Appendix 6.
Examples of text segments for each cluster in food risk corpus**

| Lexical worlds | Examples of text segments |
| --- | --- |
| *Cluster 1 Nutritional risks and scientific research* | Diabete e colesterolo, l'olio extravergine d'oliva fa da scudo per l'organismo: ecco lo studio [Diabetes and cholesterol, extra virgin olive oil acts as a shield for the body: here is the study]  Dieta vegetariana e diabete, uno studio americano la promuove [Vegetarian diet and diabetes, an American study promotes it] |
| *Cluster 2 Nutritional properties of food and substances* | patate aiutano a prevenire l’infarto, merito di vitamine e nutrienti  [potatoes help preventing heart attack, thanks to vitamins and nutrients]  le vitamine dell uva contro l'insonnia  [grape vitamins against insomnia] |
| *Cluster 3 Food products* | Frutta e verdura di Agosto, ecco i prodotti d stagione [Fruit and vegetables of August, here are the seasonal products]  Alternative naturali allo zucchero [natural alternatives to sugar] |
| *Cluster 4 Food practices at home* | Come conservare gli alimenti in frigorifero [How to store food in the fridge]  Pesce fresco, come riconoscerlo e come conservarlo prima di mangiarlo [fresh fish, how to recognize and store it before eating] |
| *Cluster 5 Educational/promotional initiatives organized by companies and institutions* | [...]l'evento organizzato in collaborazione con l'orto botanico dell'Università di Tor Vergata Roma[...] […the event organized in collaboration with the botanical garden of the University of Tor Vergata Rome…]  le famiglie coinvolte, che per una settimana e sotto il coordinamento della ricercatrice Claudia Giordano dell Università di Bologna[...] [the families involved, who for a week and under the coordination of the researcher Claudia Giordano of the University of Bologna...] |
| *Cluster 6 Environmental sustainability* | Grazie a queste iniziative e alla sensibilizzazione dei cittadini nei confronti dello spreco, i rifiuti alimentari si sono ridotti del 25% in cinque anni [Thanks to these initiatives and to the sensitization of citizens towards waste, food waste has decreased by 25% in five years]  […] le critiche all’olio di palma per non giungono solo dal punto di vista della salute ma anche sul fronte ambientale […criticisms of palm oil not only come from the health point of view but also on the environmental front] |
| *Cluster 7 Production chain* | boom del biologico, aumentano produzione e consume [organic food, production and consumption increase]  Coldiretti toscana: filiere trasparenti a garanzia dei consumatori [Coldiretti (Italian producers’ association) Tuscany: transparent supply chains to ensure consumers' safety] |
| *Cluster 8 Chemical risks and risks assessment* | Glifosato, parlamento UE nomina la commissione speciale sui pesticidi [Glyphosate, EU parliament nominates the special commission on pesticides]  Olio di palma, novità dall’ESFA [palm oil, news from the EFSA] |
| *Cluster 9 Withdrawals/recalls and seizures and alerts (Fipronil case)* | Uova contaminate: dai controlli del Ministero due lotti positivi al fipronil [Contaminated eggs: from the controls of the Ministry two lots resulted positive to fipronil]  Ritirato lotto di filetti di acciughe per istamina in eccesso  [lot of anchovy fillets withdrawn for excess histamine] |
